# Trends in oral anticoagulant choice for acute stroke patients with nonvalvular atrial fibrillation in Japan: The SAMURAI‐NVAF Study

**DOI:** 10.1111/ijs.12452

**Published:** 2015-01-12

**Authors:** Kazunori Toyoda, Shoji Arihiro, Kenichi Todo, Hiroshi Yamagami, Kazumi Kimura, Eisuke Furui, Tadashi Terasaki, Yoshiaki Shiokawa, Kenji Kamiyama, Shunya Takizawa, Satoshi Okuda, Yasushi Okada, Tomoaki Kameda, Yoshinari Nagakane, Yasuhiro Hasegawa, Hiroshi Mochizuki, Yasuhiro Ito, Takahiro Nakashima, Kazuhiro Takamatsu, Kazutoshi Nishiyama, Kazuomi Kario, Shoichiro Sato, Masatoshi Koga, SAMURAI Study Investigators, K Nagatsuka, K Minematsu, J Nakagawara, H Akiyama, K Shibazaki, K Maeda, S Shibuya, S Yoshimura, K Endo, T Miyagi, M Osaki, J Kobayashi, T Okata, E Tanaka, Y Sakamoto, H Takizawa, J Takasugi, K Tokunaga, K Homma, N Kinoshita, T Matsuki, K Higashida, M Shiozawa, H Kanai, S Uehara

**Affiliations:** ^1^ Department of Cerebrovascular MedicineNational Cerebral and Cardiovascular CenterSuitaOsakaJapan; ^2^ Department of NeurologyKobe City Medical Center General HospitalKobeHyōgoJapan; ^3^ Department of NeurologyNational Cerebral and Cardiovascular CenterSuitaOsakaJapan; ^4^ Department of Stroke MedicineKawasaki Medical SchoolKurashikiOkayamaJapan; ^5^ Department of Stroke NeurologyKohnan HospitalSendaiMiyagiJapan; ^6^ Department of NeurologyJapanese Red Cross Kumamoto HospitalKumamotoKumamotoJapan; ^7^ Departments of Neurosurgery and Stroke CenterKyorin University School of MedicineMitakaTokyoJapan; ^8^ Department of NeurosurgeryNakamura Memorial HospitalSapporoHokkaidoJapan; ^9^ Department of NeurologyTokai University School of MedicineIseharaKanagawaJapan; ^10^ Department of NeurologyNHO Nagoya Medical CenterNagoyaChūbuJapan; ^11^ Department of Neurology and Cerebrovascular MedicineNHO Kyushu Medical CenterFukuokaFukuokaJapan; ^12^ Division of NeurologyJichi Medical University School of MedicineShimotsukeTochigiJapan; ^13^ Department of NeurologyKyoto Second Red Cross HospitalKyotoHonshuJapan; ^14^ Department of NeurologySt Marianna University School of MedicineKawasakiKanagawaJapan; ^15^ Department of NeurologySouth Miyagi Medical CenterOgawaraMiyagiJapan; ^16^ Department of NeurologyTOYOTA Memorial HospitalToyotaAichiJapan; ^17^ Department of Cerebrovascular MedicineNHO Kagoshima Medical CenterKagoshimaKagoshimaJapan; ^18^ Department of NeurologyBrain Attack Center Ota Memorial HospitalFukuyamaHiroshimaJapan; ^19^ Department of NeurologyKitasato University School of MedicineSagamiharaKanagawaJapan; ^20^ Division of Cardiovascular MedicineJichi Medical University School of MedicineShimotsukeTochigiJapan; ^21^ National Cerebral and Cardiovascular Center; ^22^ Nakamura Memorial Hospital; ^23^ St Marianna University School of Medicine; ^24^ Kawasaki Medical School; ^25^ National Hospital Organization Kyushu Medical Center; ^26^ South Miyagi Medical Center

**Keywords:** acute stroke care, anticoagulation, atrial fibrillation, embolism, prevention

## Abstract

**Background:**

Large clinical trials are lack of data on non‐vitamin K antagonist oral anticoagulants for acute stroke patients.

**Aim:**

To evaluate the choice of oral anticoagulants at acute hospital discharge in stroke patients with nonvalvular atrial fibrillation and clarify the underlying characteristics potentially affecting that choice using the multicenter Stroke Acute Management with Urgent Risk‐factor Assessment and Improvement‐NVAF registry (ClinicalTrials.gov NCT01581502).

**Method:**

The study included 1192 acute ischemic stroke/transient ischemic attack patients with nonvalvular atrial fibrillation (527 women, 77·7 ± 9·9 years old) between September 2011 and March 2014, during which three nonvitamin K antagonist oral anticoagulant oral anticoagulants were approved for clinical use. Oral anticoagulant choice at hospital discharge (median 23‐day stay) was assessed.

**Results:**

Warfarin was chosen for 650 patients, dabigatran for 203, rivaroxaban for 238, and apixaban for 25. Over the three 10‐month observation periods, patients taking warfarin gradually decreased to 46·5% and those taking nonvitamin K antagonist oral anticoagulants increased to 48·0%. As compared with warfarin users, patients taking nonvitamin K antagonist oral anticoagulants included more men, were younger, more frequently had small infarcts, and had lower scores for poststroke CHADS_2_, CHA_2_ DS_2_‐VASc, and HAS‐BLED, admission National Institutes of Health stroke scale, and discharge modified Rankin Scale. Nonvitamin K antagonist oral anticoagulants were started at a median of four‐days after stroke onset without early intracranial hemorrhage. Patients starting nonvitamin K antagonist oral anticoagulants earlier had smaller infarcts and lower scores for the admission National Institutes of Health stroke scale and the discharge modified Rankin Scale than those starting later. Choice of nonvitamin K antagonist oral anticoagulants was independently associated with 20‐day or shorter hospitalization (OR 2·46, 95% CI 1·87–3·24).

**Conclusions:**

Warfarin use at acute hospital discharge was still common in the initial years after approval of nonvitamin K antagonist oral anticoagulants, although nonvitamin K antagonist oral anticoagulant users increased gradually. The index stroke was milder and ischemia‐risk indices were lower in nonvitamin K antagonist oral anticoagulant users than in warfarin users. Early initiation of nonvitamin K antagonist oral anticoagulants seemed safe.

## Introduction

Between 2009 and 2013, four novel oral anticoagulants, or in other words nonvitamin K antagonist (VKA) oral anticoagulants (NOACs) 1, were shown to be at least as effective for reducing stroke and as safe as warfarin, in particular more protective against intracranial hemorrhage (ICH) than warfarin, for patients with nonvalvular atrial fibrillation (NVAF) 2, 3, 4, 5. These NOACs have a wider therapeutic range and fewer drug and food interactions than VKAs, and were also proven to be useful for secondary stroke prevention in patients with NVAF 6, 7, 8. The effect of NOACs seems to be clearer in Asians than in non‐Asians 9, 10. Of the NOACs, dabigatran (March 2011), rivaroxaban (April 2012), and apixaban (February 2013) began to be used clinically after official approval in Japan. The sudden increase in the options for oral anticoagulants (OACs) after long years of restricted choice of VKAs alone has brought confusion in the choice of the optimal OAC for each NVAF patient. In addition, the clinical trials on NOACs excluded acute stroke patients within 14 days after onset (within seven‐days in ARISTOTLE) from enrollment 2, 3, 4, 5. Thus, the optimal timing for beginning NOACs for acute stroke or transient ischemic attack (TIA) patients has not been clear.

The Stroke Acute Management with Urgent Risk‐factor Assessment and Improvement (SAMURAI)‐NVAF Study was a prospective, multicenter, observational study designed to determine choice of anticoagulant therapy during the acute and chronic stages of ischemic stroke/TIA and short‐ and long‐term outcomes, including stroke recurrence and bleeding complications, in patients having NVAF. Eighteen Japanese stroke centers participated in the study (Supporting Information Appendix S1). The study was registered with ClinicalTrials.gov (NCT01581502) and the Japanese University Hospital Medical Information Network (UMIN) Clinical Trials Registry (UMIN000006930). In this initial report, the aim was to clarify the associations of underlying clinical characteristics and stroke/TIA features of the registered patients with choice of OACs at their hospital discharge. Another aim was to determine the timing of OAC initiation and the duration of acute hospital stay in patients on different OAC medications.

## Methods

In the SAMURAI‐NVAF Study, patients who were hospitalized (or initiated acute management at the outpatient clinic) within seven‐days after onset of ischemic stroke/TIA and were diagnosed as having NVAF between September 2011 and March 2014 were enrolled. NVAF was diagnosed on 12‐lead electrocardiogram or 24‐h or longer monitoring for atrial fibrillation (AF) detection during acute hospitalization or from previous medical documents. Patients who met the following criteria were excluded: rheumatic mitral valve disease; a history of prosthetic valve replacement or mitral valve surgical repair; active infectious endocarditis; or lack of written informed consent by patients or next of kin. All study procedures were reviewed and approved by the local Ethics Committees.

Each patient was identified by a linkable patient identification code and was registered along with clinical information via the web‐based registration system. Of the documented information, the variables listed in Table 1 were assessed in this study. The CHADS_2_ and CHA_2_DS_2_‐VASc scores as ischemic stroke risk indices and the HAS‐BLED score as a bleeding‐risk index were assessed both before and after onset of the index stroke/TIA 11, 12, 13.

**Table 1:** Underlying characteristics and stroke features of patients according to anticoagulant choice at discharge

	Total^a^ (*n* = 1192)	Warfarin (*n* = 650)	Dabigatran (*n* = 203)	Rivaroxaban (*n* = 238)	Apixaban (*n* = 25)	None (*n* = 49)	Any NOAC (*n* = 466)	*P* (W vs. NOAC)
Women	527 (44·2)	313 (48·2)	67 (33·0)	92 (38·7)	8 (32·0)	27 (55·1)	167 (35·8)	<0·001
Age, years	77·7 ± 9·9	79·1 ± 9·7	73·1 ± 8·8	75·8 ± 9·0	74·0 ± 12·0	85·0 ± 10·7	74·5 ± 9·2	<0·001
CHADS2^b^	4 [3–4]	4 [3–5]	3 [3–4]	4 [3–4]	4 [3–4]	4 [3–4]	4 [3–4]	<0·001
CHA2DS2‐VASc^b^	5 [4–6]	6 [5–6]	5 [4–6]	5 [4–6]	5 [4–6]	6 [5–6·5]	5 [4–6]	<0·001
HAS‐BLED^b^	3 [3–4]	3 [3–4]	3 [3–4]	3 [2–4]	3 [3–4]	3 [3–4]	3 [3–4]	0·002
Body weight, kg	56·3 ± 12·3	54·3 ± 12·1	61·4 ± 11·2	58·3 ± 11·5	58·5 ± 15·3	51·9 ± 11·8	59·7 ± 11·7	<0·001
Creatinine clearance, ml/min	56·6 ± 26·3	51·2 ± 25·7	71·7 ± 22·4	61·9 ± 21·8	60·8 ± 33·5	38·4 ± 22·0	66·1 ± 23·3	<0·001
Atrial fibrillation								
Unidentified^c^	466 (39·1)	227 (34·9)	88 (43·4)	105 (44·1)	7 (28·0)	24 (49·0)	200 (42·9)	0·007
Paroxysmal	434 (36·4)	210 (32·3)	87 (42·9)	99 (41·6)	10 (40·0)	18 (36·7)	196 (42·1)	<0·001
Premorbid oral anticoagulants								<0·001
Warfarin	341 (28·6)	241 (37·1)	37 (18·2)	49 (20·6)	6 (24·0)	4 (8·2)	92 (19·7)	
Dabigatran	23 (1·9)	15 (2·3)	3 (1·5)	4 (1·7)	0	1 (2·0)	7 (1·5)	
Rivaroxaban	15 (1·3)	7 (1·1)	2 (1·0)	6 (2·5)	0	0	8 (1·7)	
Stroke features								
TIA	51 (4·3)	23 (3·5)	11 (5·4)	15 (6·3)	1 (4·0)	1 (2·0)	27 (5·8)	0·073
Infarct size^d^								<0·001
Small	263 (23·7)	133 (21·6)	58 (31·2)	57 (26·8)	8 (34·8)	4 (8·7)	123 (29·1)	
Medium	534 (48·1)	277 (44·9)	110 (59·1)	116 (54·5)	14 (60·9)	14 (30·4)	240 (56·9)	
Large	314 (28·3)	207 (33·5)	18 (9·7)	40 (18·8)	1 (4·3)	28 (60·9)	59 (14·0)	
Admission NIHSS score	8 [2–18]	11 [4–20]	4 [1–8]	5 [2–14]	7 [1–14]	18 [9–24]	4 [1–12]	<0·001
NIHSS score at day 7	3 [0–12·75]	6 [1–17]	1 [0–2]	1 [0–4]	2 [0–5]	20 [5·5–27]	1 [0–3]	<0·001
Discharge mRS score	3 [1–4]	4 [1–5]	1 [0–2]	2 [1–3·25]	2 [1–3]	5 [4–5]	1 [0–3]	<0·001

Data are presented as means ± SD, medians (interquartile range), or numbers (%).

a Including 27 patients with acute hospital death.

b After onset of index stroke/transient ischemic attack (TIA).

c Unidentified prior to index stroke/TIA.

d TIA patients and those with incomplete data are excluded.

mRS, modified Rankin Scale; NIHSS, National Institutes of Health stroke scale; NOAC, nonvitamin K antagonist oral anticoagulant.

As stroke features, infarct size was defined as small when the longest diameter was ≤15 mm; as large when the infarct was larger than one‐third of the territory of the middle cerebral artery, anterior cerebral artery, posterior cerebral territory, or cerebellar hemisphere; and as medium for the others. Neurologic deficits were assessed using the National Institutes of Health stroke scale (NIHSS) score on admission and at seven‐days after onset. Functional outcome was assessed using the modified Rankin Scale (mRS) score at acute hospital discharge (or 30 days after onset, whichever occurred first; median 23 days).

Patient eligibility for anticoagulant therapy and choice of OACs were determined by each investigator. In this study, OACs chosen on the day of acute hospital discharge (or 30 days after onset, whichever occurred first) were investigated. The clinical characteristics of patients taking different OACs at discharge were compared. Trends in the choice were compared according to three 10‐month observation periods (September 2011 to June 2012, July 2012 to April 2013, and May 2013 to March 2014). In addition, the days until initiating OAC medication after stroke onset and the duration of acute hospital stay with the different OACs were evaluated.

### Statistics

Data are presented as means ± SD, median values (interquartile range), or numbers (%). Underlying characteristics and stroke features were compared using χ^2^ tests, unpaired *t‐*tests, and Wilcoxon's test, as appropriate. Trends among the three periods were compared using χ^2^ tests and one‐way factorial analysis of variance, as appropriate. Days until starting OACs and those of hospital stay were compared using Kruskal–Wallis test. Multivariate logistic regression analysis was performed to identify parameters associated with short hospital stay using the forced entry method for potential confounding factors. All statistical analyses were conducted using JMP 9·0·2 statistical software (SAS Institute, Cary, NC, USA). A *P* value of <0·05 was considered significant.

## Results

A total of 1192 patients (527 women, 77·7 ± 9·9 years old) were registered in the study. All patients were hospitalized. Of these, 820 patients (68·8%) were admitted on the day of onset, 234 (19·6%) on the next day, and 138 (11·6%) two‐days or later after onset. The underlying characteristics and stroke features of the patients are listed in Table 1. The median admission NIHSS score was 8 [interquartile range (IQR) 2–18], and the median discharge mRS score was 3 (1–4). Supporting Information Fig. S1 shows changes in CHADS_2_, CHA_2_DS_2_‐VASc, and HAS‐BLED scores before and after developing the index stroke/TIA. The median scores changed from: 2 (95% CI 1–3) to 4 (3–4, *P* < 0·001) for CHADS_2_; from 4 (3–5) to 5 (4–6, *P* < 0·001) for CHA_2_DS_2_‐VASc; and from 2 (2–3) to 3 (3–4, *P* < 0·001) for HAS‐BLED.

**Figure 1:**
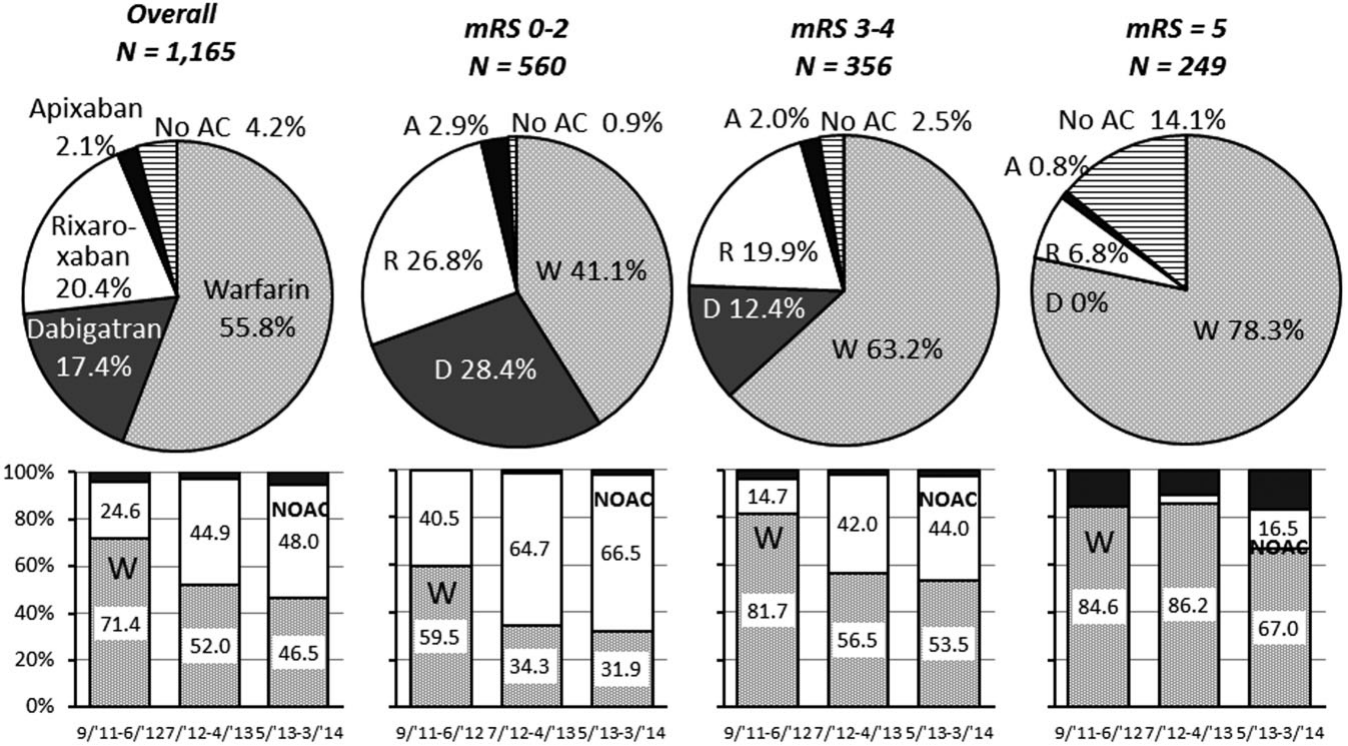
Choice of oral anticoagulants at acute hospital discharge in overall patients and in patients with different discharge mRS scores. Upper panels: percentage of oral anticoagulant use. Bottom panels: Change in percentage of warfarin and nonvitamin K antagonist oral anticoagulant (NOAC) use over the three periods. *P* < 0·001 in all. W, warfarin; D, dabigatran; R, rivaroxaban; A, apixaban; No AC, no anticoagulation.

Of the 1192 patients, 27 died during acute hospitalization; 20 died directly from stroke. In the remaining 1165 patients, warfarin was chosen as the OAC at discharge for 650 patients (55·8%), dabigatran for 203 (17·4%), rivaroxaban for 238 (20·4%), apixaban for 25 (2·1%), and OACs were not chosen for 49 (4·2%, Fig. 1). Over the three 10‐month observation periods, patients taking warfarin decreased from 71·4% to 52·0%, and finally to 46·4%; and patients taking NOACs increased from 24·6% to 44·9%, and finally to 48·1% (*P* < 0·001). Patients’ characteristics for those on warfarin and those on NOACs were compared (Table 1). NOAC users included more men, were younger, and had lower poststroke scores (CHADS_2_, CHA_2_DS_2_‐VASc, and HAS‐BLED), lower weights, and higher creatinine clearance than warfarin users. NVAF was less commonly identified prior to index stroke/TIA and was more commonly paroxysmal, and premorbid warfarin medication was less common in NOAC users. As stroke features, NOAC users more commonly had small infarcts and had lower NIHSS scores (both on admission and at seven‐days) and discharge mRS scores than warfarin users. When only the patients in the third period (May 2013 to March 2014) were assessed, ischemia‐ and hemorrhage‐risk indices were still lower, and the index stroke was still milder in NOAC users than in warfarin users (Supporting Information Table S1). In NOAC users, the creatinine clearance decreased gradually over the three periods (71·6 ± 24·2, 69·0 ± 23·4, 60·9 ± 21·7 ml/min, *P* < 0·0001).

The percentages of OAC choice differed greatly among patients with different discharge mRS scores (Fig. 1). In patients with mRS score 0–2, NOACs were more common than warfarin (58·1% vs. 41·1%). In the third period, NOAC use was more than twice as common as warfarin use (66·5% vs. 31·9%). In patients with mRS score 5, warfarin users accounted for 91·1% of any OAC users (195/214).

**Figure 2:**
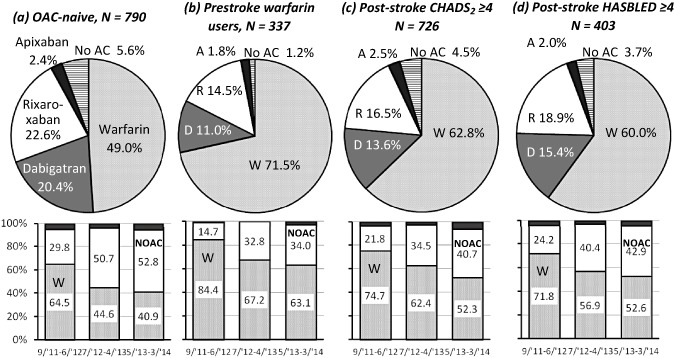
Choice of oral anticoagulants (OACs) at acute hospital discharge in prestroke OAC nonusers (a), prestroke warfarin users (b), patients with poststroke CHADS2 ≥4 (c), and those with poststroke HASBLED ≥4 (d). Upper panels: percentage of oral anticoagulant use. Bottom panels: change in percentage of warfarin and nonvitamin K antagonist oral anticoagulant (NOAC) use over the three periods. *P* < 0·001 in all. W, warfarin; D, dabigatran; R, rivaroxaban; A, apixaban; No AC, no anticoagulation.

Of the 1165 patients, 790 (67·8%) did not receive any OAC prior to the index stroke/TIA (Fig. 2a). Of these, 387 (49·0%) chose warfarin, and 359 (45·4%) chose NOACs at hospital discharge. Over the three periods, patients taking NOACs increased from 29·8% to 50·7%, and finally to 52·8% (*P* < 0·001). Of the remaining 375 OAC‐experienced patients, 337 were warfarin users (Fig. 2b); the median international normalized ratio (INR) of prothrombin time on admission was 1·34 (IQR 1·14–1·66). Of these, 241 (71·5%) with median admission INR of 1·39 (1·14–1·75) resumed warfarin, and 92 (27·3%) with median INR of 1·30 (1·12–1·50) were changed to NOAC.

Warfarin was chosen for 456 (62·8%) of 726 patients with poststroke CHADS_2_ ≥ 4, and 242 (60·0%) of 403 patients with poststroke HASBLED ≥ 4; the percentage of warfarin users decreased gradually, and that of NOAC users increased gradually over the three periods in both groups (*P* < 0·001 for both, Fig. 2c,d).

**Figure 3:**
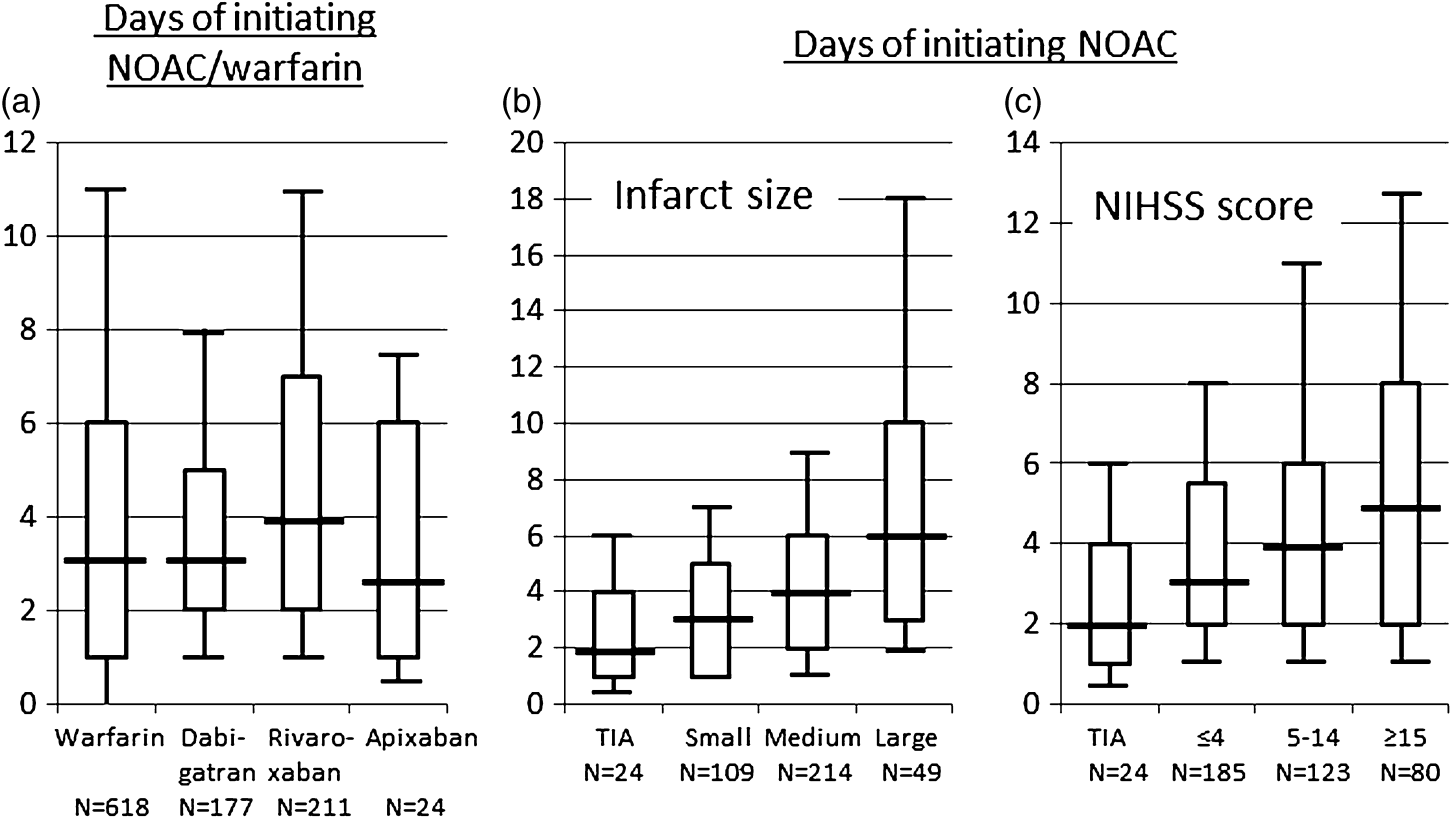
Days prior to initiating oral anticoagulants (OACs). (a) Days of initiating OACs after onset of index stroke/TIA. Eighty‐six patients who changed OACs during acute hospitalization were excluded. (b) Days of initiating nonvitamin K antagonist OACs (NOACs) according to infarct size. (c) Days of initiating NOACs according to initial neurological severity. Boxes represent interquartile range. Lines across box indicate median values. Whiskers represent 10 percentile and 90 percentile values. NIHSS, National Institutes of Health stroke scale; TIA, transient ischemic attack.

Figure 3a shows the days until starting OAC medication. The median days of initiation after stroke/TIA onset were three‐days for warfarin and dabigatran, four‐days for rivaroxaban, and 2·5 days for apixaban; the median was four‐days (IQR two to six‐days) for any NOAC. The median days of initiating NOACs were two‐days in TIA patients, three‐days in small‐size stroke patients, four‐days in medium‐size stroke patients, and six‐days in large‐size stroke patients (Fig. 3b, *P* < 0·01); they were three‐days in patients with admission NIHSS score ≤4, four‐days in those with a score of 5 to 14, and five‐days in those with a score ≥15 (Fig. 3c, *P* < 0·01). As compared with patients starting NOACs at ≥four‐days, NVAF was more commonly identified prior to index stroke/TIA and less commonly paroxysmal, infarcts were smaller, and scores of NIHSS (both on admission and at seven‐days) and of discharge mRS were lower in patients starting NOACs within three‐days (Supporting Information Table S2). One patient who started rivaroxaban two‐days after stroke onset developed gastrointestinal bleeding seven‐days later. None of the NOAC users developed ICH prior to discharge.

The median duration of acute hospital stay (or 30 days after onset, whichever occurred first) was 27 days in warfarin users, 16 days in dabigatran users, 18·5 days in rivaroxaban users, and 20 days in apixaban users; it was 17 days (IQR 12–26 days) for any NOAC users. After adjustment for gender, age, and initial NIHSS score, NOAC use was independently associated with acute hospital stay within 20 days (OR 2·44, 95% CI 1·86–3·22, Table 2). After adjustment for gender, age, and discharge mRS score, NOAC use was also independently associated with acute hospital stay within 20 days (OR 1·92, 95% CI 1·44–2·56).

**Table 2:** Multivariate‐adjusted association of anticoagulant choice with hospital stay within 20 days

	OR	95% CI	*P*
Model 1			
Women (vs. men)	0·73	0·55–0·97	0·031
Age, per 10 years	1·16	1·00–1·34	0·052
Initial NIHSS score, per 1 point	0·91	0·89–0·92	<0·001
NOAC (vs. warfarin)	2·46	1·87–3·24	<0·001
Model 2			
Women (vs. men)	0·79	0·59–1·06	0·112
Age, per 10 years	1·44	1·23–1·69	<0·001
Discharge mRS score, per 1 point	0·53	0·48–0·58	<0·001
NOAC (vs. warfarin)	1·92	1·44–2·56	<0·001

NIHSS, National Institutes of Health stroke scale; NOAC, nonvitamin K antagonist oral anticoagulant; mRS, modified Rankin Scale.

## Discussion

In this prospective observational study, several major findings related to the trends of OAC choice for NVAF patients with acute ischemic stroke/TIA over 31 months during which three NOACs were approved for clinical use in Japan were identified. The first finding was that warfarin use at acute hospital discharge was still common in patients overall, although NOAC users increased gradually and exceeded warfarin users in the last 11 months. Second, the index stroke was milder and the ischemia‐risk indices were lower in patients taking NOACs than in those taking warfarin at discharge. In particular, NOACs were prevalent for independent patients, corresponding to discharge mRS scores 0–2, and warfarin was overwhelmingly more common for patients with mRS score 5. Third, prior OAC nonusers relatively often chose NOACs at discharge as compared with prior warfarin users. In contrast, 72% of prior warfarin users resumed anticoagulation with warfarin, although the stroke/TIA had not been prevented using this agent. Fourth, NOAC therapy was initiated at a median four‐days after stroke/TIA onset, with small infarct and mild neurological symptoms being associated with early initiation of NOACs. NOAC users did not develop ICH during acute hospitalization. Finally, NOAC use was independently associated with acute hospital stay after adjustment for initial NIHSS scores or discharge mRS scores.

The European Society of Cardiology guidelines advocate that, when an OAC is recommended for NVAF patients, one of the NOACs should be considered instead of adjusted‐dose warfarin 14. The Japanese guidelines also referred to the advantages of NOAC use in patients with CHADS_2_ ≥ 2 15. On the other hand, in the AHA/ACC/HRS guidelines, the levels of evidence were higher for warfarin than for NOACs 16. As described above, dabigatran was approved in clinical use several months before study initiation, and rivaroxaban and apixaban were approved during the study period in Japan. As it is not allowed to prescribe new drugs for more than 14 days at outpatient clinics within a year after approval in Japan, it usually takes time for the new drug use to increase. This might be a reason that NOAC use was relatively infrequent, and apixaban users were especially infrequent in this study.

Based on the results of a meta‐analysis of the trials, NOACs offer better efficacy than warfarin for elderly (≥75 years old) and high CHADS_2_ score (3–6) patients 17. However, the present NOAC users were younger, had lower CHADS_2_ scores, and had obviously milder strokes than warfarin users. NOAC use accounted for around three‐fifths of patients with discharge mRS scores 0–2, whereas warfarin use accounted for more than nine‐tenths of anticoagulant users with discharge mRS score 5. There are a few possible reasons for the uncommon NOAC use for more severe stroke patients. The first reason might be economic issues. In nursing homes and chronic hospitals, it is often difficult to offer expensive drugs due to financial or insurance limitations; thus, NOACs might not be chosen in acute hospitals for patients who are less likely to be directly discharged home. Severe stroke patients might lose their job or become financially disadvantaged and stay away from expensive NOACs. The second possible reason is dysphagia. Dabigatran cannot be crushed and is not suitable for dysphagic patients. Although tablets of rivaroxaban and apixaban may be effective after crushing, the method has not been in wide use as for warfarin 18. In addition, severe stroke patients often have advanced renal insufficiency 19. Because the NOAC with low renal excretion was approved in the later periods, the mean creatinine clearance of NOAC users gradually decreased over the three periods.

Physicians relatively often chose NOACs for OAC‐naïves. However, three‐fourths of prior warfarin users were put back on warfarin after its failure to prevent stroke/TIA. As most of prior warfarin users showed subtherapeutic INR on admission, many physicians might prefer to continue warfarin with optimal dose adjustment rather than change to NOACs. Nevertheless, it may be difficult to maintain the optimal INR range for patients whose warfarin intensity was once underpowered, because the intensity depends on many factors other than the efforts of physicians.

The optimal timing for initiation of oral anticoagulation has not been established. In the AHA/ASA guidelines, it is regarded as reasonable to initiate OACs within 14 days after onset 20. The European Heart Rhythm Association Practical Guide introduces personalized recommendation using the 1–3–6–12 day rule depending on stroke severity 21. In the present cohort, even severe patients with an initial NIHSS score ≥15 started NOACs at a median five‐days after onset. ICH did not occur in NOAC users during acute hospitalization. Thus, early NOAC initiation may be safe. In a study of 41 patients starting NOACs at a median of two‐days after onset, symptomatic ICH was not identified 22.

In Japan, hospital stays for acute stroke are usually longer than in other countries partly because of the differences in the medical insurance systems and partly because recovery rehabilitation therapy is also performed in some acute hospitals. Patients taking NOACs had median stays that were 10 days shorter than warfarin users, and NOAC use was related to shorter stay, independently of initial neurological severity or independence at discharge. The main cause appears to be the difference in the duration for reaching pharmacologically steady state between warfarin and NOACs.

This study has some limitations. First, it was an observational study and the choice of OACs was determined by each investigator. Thus, the underlying characteristics of the OAC users differed greatly. Second, the findings regarding OAC choice and length of hospitalization may not be generalizable to countries where hospital stays are much shorter and warfarin dosing is often stabilized on an outpatient basis. Third, this study did not assess the occurrence of ischemic and hemorrhagic events after registration. This theme will be discussed elsewhere when the observations are completed.

In conclusion, the choice of OAC for stroke/TIA patients with NVAF during the initial years of NOACs in Japan was evaluated. NOAC use increased gradually during the study period, but NOACs were mainly taken by younger patients with milder strokes. The present results might not be innovative but depict the cautious clinical approach of neurologist to the novel pharmacotherapy. A unique feature of this cohort was the relatively high ischemia‐ and hemorrhage‐risk indices after the index stroke/TIA. Using this cohort, we are continuing to explore event occurrence and related characteristics.

## Supporting information


**Fig. S1.** Changes in CHADS_2_, CHA_2_DS_2_‐VASc, and HAS‐BLED scores between before and after onset of index stroke or transient ischemic attack.
**Table S1.** Underlying characteristics and stroke features of patients in the third period (May 2013–March 2014) according to anticoagulant choice at discharge.
**Table S2.** Underlying characteristics and stroke features of patients starting nonvitamin K antagonist oral anticoagulants (NOACs) within three‐days after onset and those initiating NOACs at four‐days or later.

**Appendix S1.** Participating sites and investigators.
