# Supplementary material for: Trends in oral anticoagulant choice for acute stroke patients with nonvalvular atrial fibrillation in Japan: The SAMURAI‐NVAF Study
Source: Int J Stroke. 2015 Jan 12;10(6):836–42. doi: 10.1111/ijs.12452 (PMC4964913; doi:10.1111/ijs.12452)

**APPENDIX**

**Participating Sites and Investigators**

****Chief Investigator**:** K Toyoda (National Cerebral and Cardiovascular Center)

****Investigators and Institutions****:

K Todo (Kobe City Medical Center General Hospital)

K Kamiyama, J Nakagawara (Nakamura Memorial Hospital)

E Furui (Kohnan Hospital)

Y Shiokawa (Kyorin University School of Medicine)

Y Hasegawa, H Akiyama (St Marianna University School of Medicine)

S Okuda (National Hospital Organization Nagoya Medical Center)

K Kimura, K Shibazaki (Kawasaki Medical School)

Y Okada, K Maeda (National Hospital Organization Kyushu Medical Center)

K Kario, T Kameda (Jichi Medical University School of Medicine)

H Mochizuki, S Shibuya (South Miyagi Medical Center)

Y Ito (TOYOTA Memorial Hospital)

Y Nagakane (Kyoto Second Red Cross Hospital)

K Takamatsu (Brain Attack Center Ota Memorial Hospital)

T Terasaki (Japanese Red Cross Kumamoto Hospital)

T Nakashima (National Hospital Organization Kagoshima Medical Center)

S Takizawa (Tokai University School of Medicine)

K Nishiyama (Kitasato University School of Medicine)

****Central Office**** (National Cerebral and Cardiovascular Center)**:**

S Arihiro, M Koga, H Yamagami, S Sato, S Yoshimura, K Endo, T Miyagi, M Osaki, J Kobayashi, T Okata, E Tanaka, Y Sakamoto, H Takizawa, J Takasugi, K Tokunaga, K Homma, N Kinoshita, T Matsuki, K Higashida, M Shiozawa, H Kanai, S Uehara

Supervisory Advisor: K Nagatsuka, K Minematsu (National Cerebral and Cardiovascular Center)

**Supplemental Table 1.**

Underlying characteristics and stroke features of patients in the third period (May 2013 – March 2014) according to anticoagulant choice at discharge

|  | Warfarin  (n=188) | Any NOAC  (n=194) | P |
| --- | --- | --- | --- |
| Women | 91 (48.4) | 70 (36.1) | 0.015 |
| Age, y | 79.5±9.7 | 75.7±9.0 | <0.001 |
| CHADS2 * | 4 [3-5] | 4 [3-4] | <0.001 |
| CHA2DS2-VASc * | 6 [5-6] | 5 [4-6] | <0.001 |
| HAS-BLED * | 3 [3-4] | 3 [2-4] | 0.013 |
| Body weight, kg | 53.8±11.8 | 58.0±11.6 | <0.001 |
| Creatinine clearance, mL/min | 47.6±23.9 | 60.9±21.7 | <0.001 |
| *Atrial fibrillation* |  |  |  |
| Unidentified^†^ | 62 (33.0) | 82 (42.3) | 0.061 |
| Paroxysmal | 54 (28.7) | 82 (42.3) | 0.006 |
| *Premorbid oral anticoagulants* |  |  | <0.001 |
| Warfarin | 65 (34.6) | 35 (18.0) | 0.003 |
| Dabigatran | 2 (1.1) | 3 (1.6) |  |
| Rivaroxaban | 5 (2.7) | 6 (3.1) |  |
| *Stroke features* |  |  |  |
| TIA | 8 (4.3) | 15 (7.7) | 0.153 |
| Infarct size |  |  | <0.001 |
| Small | 31 (17.6) | 43 (25.3) |  |
| Medium | 78 (44.3) | 95 (55.9) |  |
| Large | 67 (38.1) | 32 (18.8) |  |
| Admission NIHSS score | 11 [3-20] | 4 [2-13] | <0.001 |
| NIHSS at day 7 | 6 [1-17.75] | 1 [0-4] | <0.001 |
| Discharge mRS score | 4 [2-5] | 2 [1-3] | <0.001 |

Data are presented as means±SD, medians (interquartile range), or numbers (%).

* after onset of index stroke/transient ischemic attack (TIA), †unidentified prior to index stroke/TIA

mRS: modified Rankin Scale, NIHSS: National Institutes of Health stroke scale

NOAC: non-vitamin K antagonist oral anticoagulant

**Supplemental Table 2.**

Underlying characteristics and stroke features of patients starting non-vitamin K antagonist oral anticoagulants (NOACs) within 3 days after onset and those initiating NOACs at 4 days or later

|  | ≤ 3 days  (n=205) | ≥ 4 days  (n=207) | P |
| --- | --- | --- | --- |
| Women | 72 (35.1) | 81 (39.1) | 0.400 |
| Age, y | 74.2±9.4 | 74.9±9.4 | 0.431 |
| CHADS2 * | 3 [3-4] | 4 [3-4] | 0.317 |
| CHA2DS2-VASc * | 5 [4-6] | 5 [4-6] | 0.107 |
| HAS-BLED * | 3 [3-4] | 3 [2-4] | 0.657 |
| Body weight, kg | 59.9±12.3 | 58.9±11.1 | 0.352 |
| Creatinine clearance, mL/min | 65.8±22.9 | 65.5±24.1 | 0.894 |
| *Atrial fibrillation* |  |  |  |
| Unidentified† | 76 (37.1) | 101 (48.8) | 0.016 |
| Paroxysmal | 72 (35.1) | 100 (48.8) | 0.005 |
| Premorbid warfarin | 41 (20.0) | 28 (13.5) | 0.078 |
| *Stroke features* |  |  |  |
| TIA | 16 (7.8) | 8 (3.9) | 0.088 |
| Infarct size‡ |  |  | 0.002 |
| Small | 64 (35.6) | 45 (23.4) |  |
| Medium | 102 (56.7) | 112 (58.3) |  |
| Large | 14 (7.8) | 35 (18.2) |  |
| Admission NIHSS score | 4 [1-8] | 6 [2-14] | <0.001 |
| NIHSS at day 7 | 0 [0-2] | 1 [0-6] | <0.001 |
| Discharge mRS score | 1 [0-2] | 2 [1-4] | <0.001 |

Data are presented as means±SD, medians (interquartile range), or numbers (%).

†unidentified prior to index stroke/transient ischemic attack (TIA),

‡TIA patients and those with incomplete data are excluded.

mRS: modified Rankin Scale, NIHSS: National Institutes of Health stroke scale

**Supplemental Figure 1.**

Changes in CHADS_2_, CHA_2_DS_2_-VASc, and HAS-BLED scores between before and after onset of index stroke or transient ischemic attack


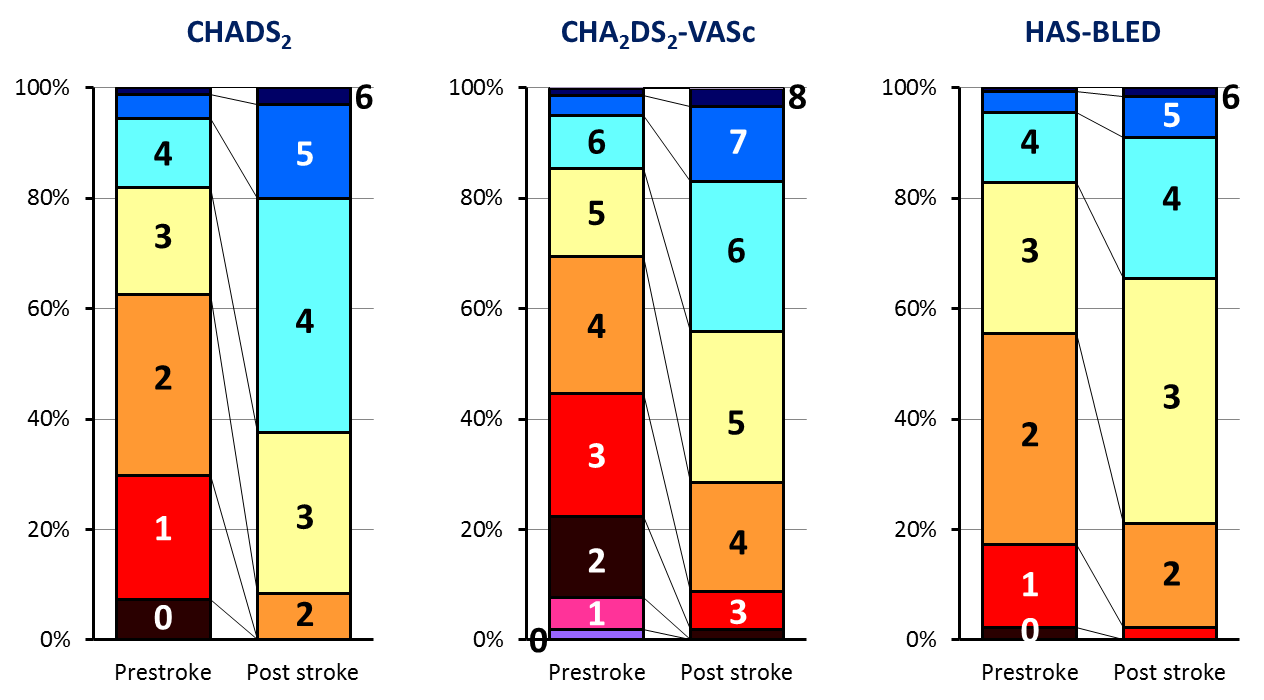

Supplement: Supplementary file 1 — Fig. S1. Changes in CHADS2, CHA2DS2‐VASc, and HAS‐BLED scores between before and after onset of index stroke or transient ischemic attack. Table S1. Underlying characteristics and stroke features of patients in the third period (May 2013–March 2014) according to anticoagulant choice at discharge. Table S2. Underlying characteristics and stroke features of patients starting nonvitamin K antagonist oral anticoagulants (NOACs) within three‐days after onset and those initiating NOACs at four‐days or later. [file IJS-10-836-s001.docx]
