# Supplementary material for: Trends in oral anticoagulant choice for acute stroke patients with nonvalvular atrial fibrillation in Japan: The SAMURAI‐NVAF Study
Source: Int J Stroke. 2015 Jan 12;10(6):836–42. doi: 10.1111/ijs.12452 (PMC4964913; doi:10.1111/ijs.12452)
Supplement: Supplementary file 2 — Appendix S1. Participating sites and investigators. [file IJS-10-836-s002.doc]

**APPENDIX**

**Participating Sites and Investigators**

**Chief Investigator**:**** K Toyoda (National Cerebral and Cardiovascular Center)

**Investigators and Institutions**:

K Todo (Kobe City Medical Center General Hospital)

K Kamiyama, J Nakagawara (Nakamura Memorial Hospital)

E Furui (Kohnan Hospital)

Y Shiokawa (Kyorin University School of Medicine)

Y Hasegawa, H Akiyama (St Marianna University School of Medicine)

S Okuda (National Hospital Organization Nagoya Medical Center)

K Kimura, K Shibazaki (Kawasaki Medical School)

Y Okada, K Maeda (National Hospital Organization Kyushu Medical Center)

K Kario, T Kameda (Jichi Medical University School of Medicine)

H Mochizuki, S Shibuya (South Miyagi Medical Center)

Y Ito (**TOYOTA** Memorial Hospital)

Y Nagakane (Kyoto Second Red Cross Hospital)

K Takamatsu (Brain Attack Center Ota Memorial Hospital)

T Terasaki (Japanese Red Cross Kumamoto Hospital)

T Nakashima (National Hospital Organization Kagoshima Medical Center)

S Takizawa (**Tokai** University School of Medicine)

K Nishiyama (**Kitasato** University School of Medicine)

**Central Office** (National Cerebral and Cardiovascular Center)****:****

S Arihiro, M Koga, H Yamagami, S Sato, S Yoshimura, K Endo, T Miyagi, M Osaki, J Kobayashi, T Okata, E Tanaka, Y Sakamoto, H Takizawa, J Takasugi, K Tokunaga, K Homma, N Kinoshita, T Matsuki, K Higashida, M Shiozawa, H Kanai, S Uehara

Supervisory Advisor: K Nagatsuka, K Minematsu (National Cerebral and Cardiovascular Center)
